# Influence of PEEK Coating on Hip Implant Stress Shielding: A Finite Element Analysis

**DOI:** 10.1155/2016/6183679

**Published:** 2016-03-14

**Authors:** Jesica Anguiano-Sanchez, Oscar Martinez-Romero, Hector R. Siller, Jose A. Diaz-Elizondo, Eduardo Flores-Villalba, Ciro A. Rodriguez

**Affiliations:** ^1^Escuela de Ingeniería y Ciencias, Tecnológico de Monterrey, Avenida Eugenio Garza Sada 2501, 64849 Monterrey, Mexico; ^2^Escuela de Medicina, Tecnológico de Monterrey, Avenida Eugenio Garza Sada 2501, 64849 Monterrey, Mexico

## Abstract

Stress shielding is a well-known failure factor in hip implants. This work proposes a design concept for hip implants, using a combination of metallic stem with a polymer coating (polyether ether ketone (PEEK)). The proposed design concept is simulated using titanium alloy stems and PEEK coatings with thicknesses varying from 100 to 400 *μ*m. The Finite Element analysis of the cancellous bone surrounding the implant shows promising results. The effective von Mises stress increases between 81 and 92% for the complete volume of cancellous bone. When focusing on the proximal zone of the implant, the increased stress transmission to the cancellous bone reaches between 47 and 60%. This increment in load transferred to the bone can influence mineral bone loss due to stress shielding, minimizing such effect, and thus prolonging implant lifespan.

## 1. Introduction

The number of total hip arthroplasties (THA) operations is increasing, reaching more than one million procedures worldwide per year. This technique is a useful treatment option for osteoarthritis and rheumatoid arthritis on the hip joint, allowing the patients to regain pain-free mobility [1]. Although THA is considered a successful procedure, recent projections indicate the number of revision surgeries is expected to increase by 137% in the next 15 years [2]. This is a major problem due to the pain and high costs caused to the patient, together with less favorable results compared to the first procedure, mainly because of the damage cause to the remaining bone after the THA. Hip implants are designed to last for at least 20 years, but their lifespan has reduced by several problems. One of the most commonly recorded indications for revision surgery is aseptic loosening, where stress shielding is a principal factor [3].

According to Wolff's law, bones adapt to the mechanical load they receive. When a person is more active in a specific part of the body, more bone is added to strengthen the tissue, and conversely, if a bone stops receiving load for a prolonged time, the mass of the tissue decreases and bone is lost. Once the hip replacement is conducted, the load is carried mainly by the implant itself and not by the femur. This phenomenon is due to a mismatch in stiffness between the hip implant and femur (almost 10 times higher in implant), with variations related to natural physiological conditions [4]. An insufficient load transfer between bone and implant leads to mineral bone lost and thus to lack of contact between the bone and femur. This effect is known as stress shielding.

The research literature shows two approaches to tackle stress shielding in hip implants: design and/or materials. Several studies have focused on changing the geometry of the hip implant in order to reduce the stress shielding effect [5–8]. Joshi et al. propose a new design and proximal fixation method to reduce stress shielding [5]. Gross and Abel use numerical analysis to show the benefits of using a hollow hip implant design [6]. In order to understand stress shielding, Boyle and Kim analyzed commercially available hip implants with consideration of microlevel bone remodeling [7]. Hirata et al. show that hip implant geometry plays a role in stress shielding, measuring the bone mineral density in patients over a period of one year [8]. The proposed hip implant concept in the study reported here is not based on a geometric approach.

Similar to geometric approach, the literature reports several studies that focus on biomimetic materials for hip implants in order to reduce stress shielding. Bougherara et al. proposed a hip implant based on polymeric composite and a hydroxyapatite-based coating [9]. Oshkour et al. propose a functionally graded hip implant based on stainless steel, titanium alloy, and hydroxyapatite. The simulation results show improvements in the stress shielding [3]. More recently, Tavakkoli Avval et al. showed the benefits of polymeric composite hip implants by conducting a coupled simulation of the bone-implant interaction biomechanics and the bone regeneration process [10].

The study reported here proposes a design concept that combines a metallic stem with an engineered polymer coating. This approach has not been reported in the relevant literature. The proposed polymer has Young's modulus similar to the bone, facilitating load transmission, and therefore, reducing the stress shielding effect. Finite Element analysis provides preliminary validation of the proposed hip implant design concept.

## 2. Materials and Methods

### 2.1. Hip Implant Design Concept

**Figure 1:**
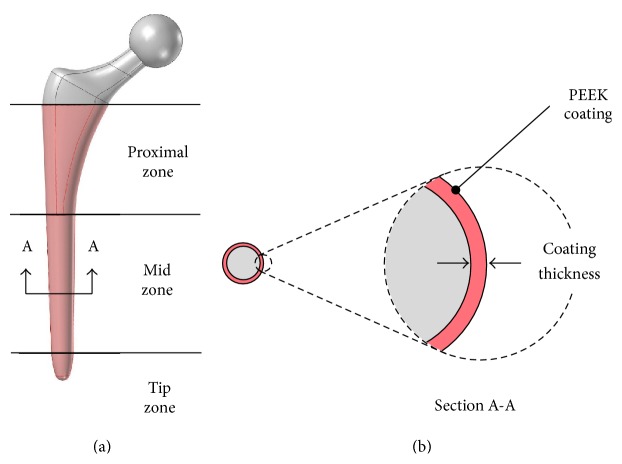
(a) Three-dimensional model for the hip implant coated with PEEK, with proposed coating location indicated in red. (b) Cross section showing the proposed PEEK coating.

The proposed design concept builds on existing technology for press-fit hip implants (see Figure 1). The stem is made out of medical grade of titanium alloy Ti-6Al-4V. The selected material for the coating is polyether ether ketone (PEEK), a well-known biocompatible polymer, used in orthopedic, spine, and dental implants. PEEK and polyaryl ether ketones (PAEKs) had been used as biomaterials since the 1980s [11], due to their structure that confers outstanding chemical resistance, inertness, and thermal stability for* in vivo* conditions. Additionally, for the purpose of this study, PEEK has Young's modulus comparable to that of the bone. Therefore, there is potential to reduce the stiffness mismatch between the femur and the hip implant.

This work investigates how a PEEK coating on a titanium alloy hip implant stem could improve the effective von Mises stress distribution on the femur, comparing a model with uncoated condition to models that have coatings with different thicknesses. An increase in effective von Mises stress values is expected, reducing stress shielding on the femur.

The coating is derived from a hypothetical manufacturing process. The coating thickness is assumed to be uniformly distributed along the surface of the implant stem, starting at the height of lesser trochanter. Figure 1 shows the PEEK coating representation and the modified Gruen zones with proximal, mid, and distal portions [7, 12].

Four different coating thicknesses were analyzed (100, 200, 300, and 400 *μ*m) and compared to the uncoated condition. These thicknesses were chosen in accordance with the standard values for electrophoretic deposition technique (EPD) [13], a method that allows fair PEEK coatings. The geometry of the hip implant is exactly the same for all simulations. The thickness portion of the coating is taken from the cancellous bone with the purpose of mimicking, the THA procedure (see Figure 1).

### 2.2. Geometric Modeling and Meshing

The standard “Sawbones” Pacific Research Labs Inc. model was used as starting point for the femur [14]. That model modified to create a new geometry of the femur after the THA procedure, on the* PTC Creo*© software. A series of parallel sketches were used in order to create a solid geometry with the least amount of sharp edges and therefore facilitate the discretization process. In addition, computerized models of the hip implant and the coating were developed.

Implant model was based on a typical metal-on-metal design. In order to facilitate the analysis, the implant geometry is a simplified version of a commercial implant such as Biomet Bi-Metric®.

**Figure 2:**
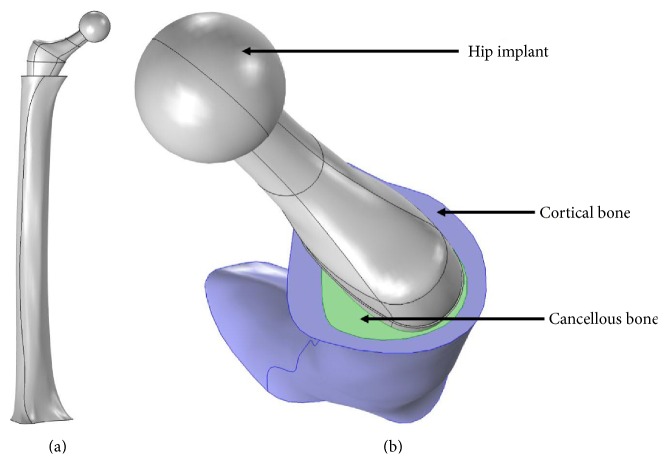
(a) FE model of femur with boundary conditions: 3,000 N in applied load at the femoral implant head and a fixation constraint at the distal end. (b) Top view of the model showing the hip implant, cortical and cancellous bone sections.

Figure 2 shows the complete assembled geometry of the implant, femur, and coating. The coating is assumed to be uniform and perfectly bonded to the stem of the hip implant.

In order to perform structural analysis by Finite Element Method (FEM), the assembly geometry was imported into* COMSOL Multiphysics*© software. The computer model was discretized with different global and local sizes. Due to the complexity of the geometry, tetrahederal elements were necessary. Finally, in order to assure numerical convergence, the most suitable mesh for the study consisted of 1,090,944 tetrahedral elements, with 0.1 and 15.4 mm as the minimum and maximum element size, respectively.

### 2.3. Material Properties and Boundary Conditions

Four different types materials were considered during numerical analysis: cancellous bone, cortical bone, PEEK coating, and hip implant. All materials are considered to be isotropic based on the part properties derived from common manufacturing techniques such as casting [15] and EPD process for the coating [16]. The hip implant material is of medical grade Ti-6Al-4V.

Mechanical properties of biological tissues vary according to factors such as age, gender, race, and other factors. Average values for cancellous and cortical bone are used [17–19]. The properties for the PEEK 150 XF (polymer used for coating) were obtained from the Victrex data sheet (http://www.victrex.com/).  Table 1 shows a summary of the mechanical properties used in the numerical analysis.

The femur was rigidly fixed at the distal end. The assembly is set to form a union, in order to simulate complete interdigitating of cortical bone, cancellous bone, and PEEK coating. A vertical load of 3,000 N is applied to the femoral implant head, representing 4 times the body weight of a 75 kg patient [17].

## 3. Results

The results are given in terms of effective von Mises stresses as experienced by the cancellous bone around the hip implant, according to suggested criteria used in previous studies [20]. The three regions mentioned before (proximal, mid, and tip) are used to analyze the change in stress transmitted to the femur. Each of these zones is evaluated independently, comparing a mean value of von Mises stress in the coated with uncoated condition.

### 3.1. Surface Analysis

*COMSOL Multiphysics* software allows the analysis of von Mises stresses by selecting surfaces and associating nodes on the mesh. The internal surfaces of the femur in contact with the hip implant were divided into proximal, mid, and tip zones. These zones required a different number of nodes: proximal (6,811 nodes), mid (6,291 nodes), and tip (3,277 nodes).

**Figure 3:**
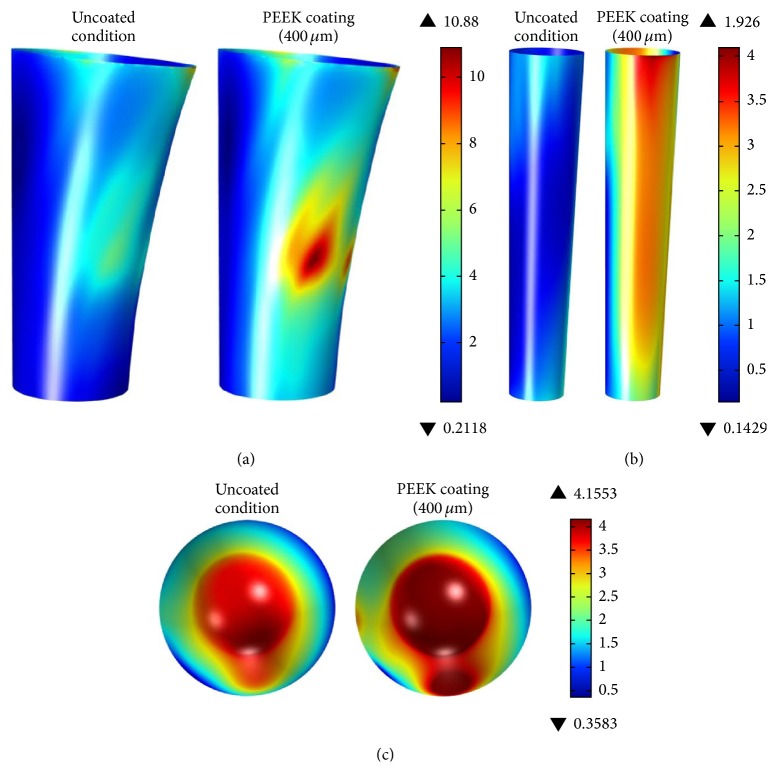
Effective von Mises stress [MPa] at cancellous bone for (a) proximal zone (lateral view), (b) mid zone (lateral view), and (c) tip zones (top view) (uncoated condition on left side and coated condition on the right side).

Figure 3 shows the Finite Element analysis results for effective von Mises stresses in the proximal, mid, and distal zones of the cancellous bone. The comparison of uncoated versus coated conditions (400 *μ*m) shows a significant increase in the transmitted stress to the femur. For the coated condition, stress is almost uniformly distributed in all lateral surfaces, except for a peak zone with a stress value of 10.79 MPa.

In the mid zone (see Figure 3(b)), the difference in stress distribution is more significant compared to the proximal and tip zones. In the case of the tip zone analysis the increase in stress distribution is moderate.

**Figure 4:**
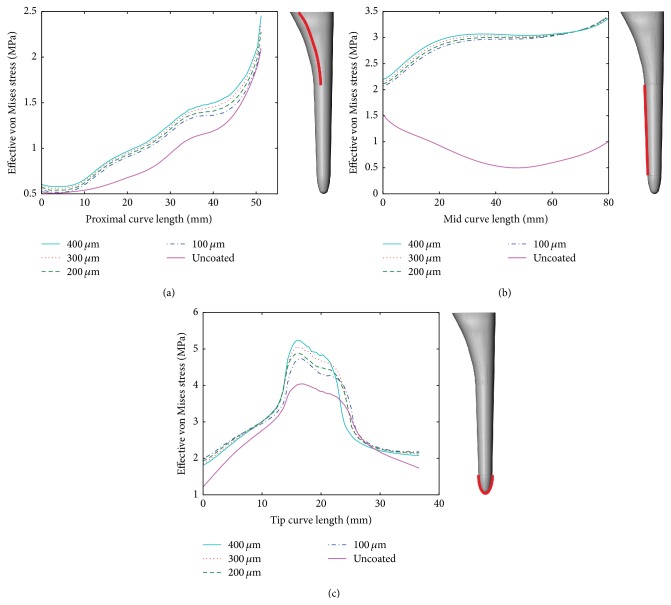
Effective von Mises stress [MPa] distribution at the cancellous bone for a curve along the surface: (a) proximal zone, (b) mid zone, and (c) tip zone (solid line: uncoated conditions, dotted lines: 100 *μ*m, 200 *μ*m, 300 *μ*m, and 400 *μ*m coatings).

Figure 4 shows the stress values for a curve along each of the zones proposed in this study. Each point represents a node along the curve of analysis. The starting point with length 0 mm is always at the top of the selected zone.

Figure 4(a) shows the effective von Mises stress on a single curve along the proximal zone. As the length increases, the effect of the coating is more noticeable. It is clear that as the coating thickens higher loads are transferred to the femur and, consequently, less stress shielding will be presented. The difference in stress transmission for a coating thickness of 400 *μ*m versus 100 *μ*m is only significant at a length between 35 and 50 mm. Therefore, for the purpose of producing a workable electrophoresis coating, the thickness can be maintained below 400 *μ*m.

Figures 4(b) and 4(c) present the results for mid and tip zone, respectively. The mid zone shows the best results in terms of load transferred to cancellous bone. In Figure 4(b) the behavior of stress distribution changes across the curve length, comparing the uncoated and the coated condition. After 60 mm all lines for each coating thickness almost overlap. For the tip zone, Figure 4(c), values of effective von Mises are higher here than in any other zone of the bone. In this case, the curve of analysis goes around the tip of the implant (see Figure 4(c)).

**Figure 5:**
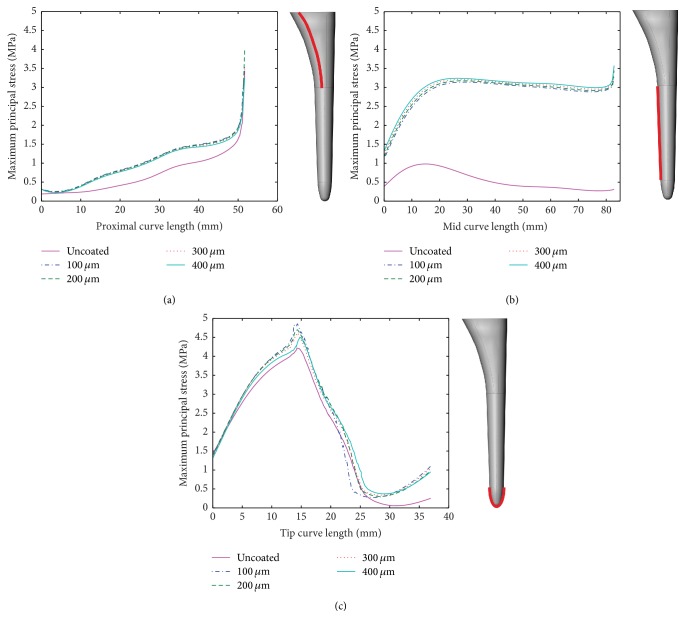
Maximum principal stress (compressive stress) [MPa] distribution at the cancellous bone for a curve along the surface: (a) proximal zone, (b) mid zone, and (c) tip zone (solid line: uncoated conditions, dotted lines: 100 *μ*m, 200 *μ*m, 300 *μ*m, and 400 *μ*m coatings).

For comparison, Figure 5 shows the analysis based on maximum principal stress. Similar to the analysis based on effective von Mises stress, the coating shows better transmission of the load to the cancellous bone.

**Figure 6:**
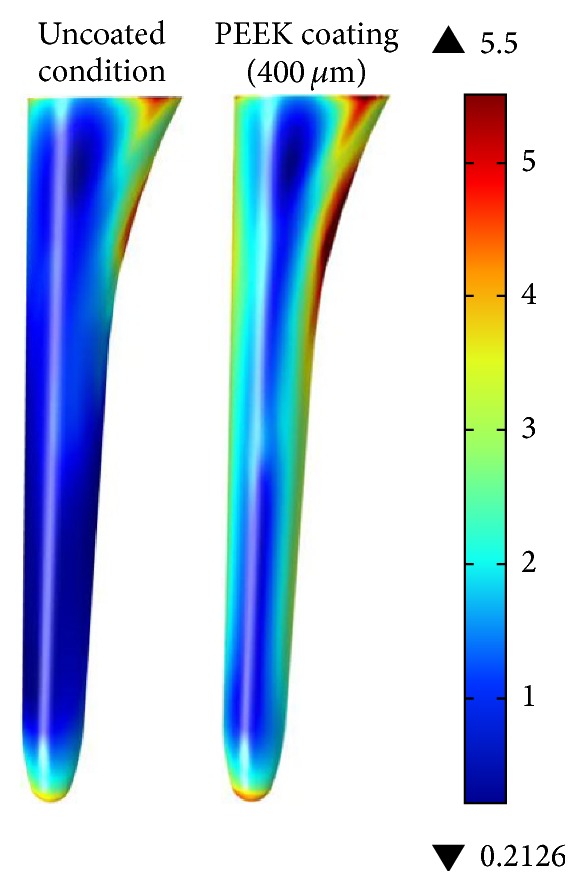
Effective von Mises stress [MPa] at cancellous bone for complete surface (uncoated condition on left side and 400 *μ*m coating on the right side).

Figure 6 shows the complete surface analysis for the cancellous bone. The results show a clear increase of stress in the lateral faces of the bone with coated condition (red zones). Under the PEEK coating of 400 *μ*m, the highest stress at the cancellous bone is 5.5 MPa.

### 3.2. Volumetric Analysis of Cancellous Bone

It is important to analyze each zone individually since proximal and mid zones are where higher stress shielding values are reported in the research literature. The current study reports consistent results, where the proximal and mid zones have a more significant role in terms of load transferred to bone, even with the 100 *μ*m coating.

**Figure 7:**
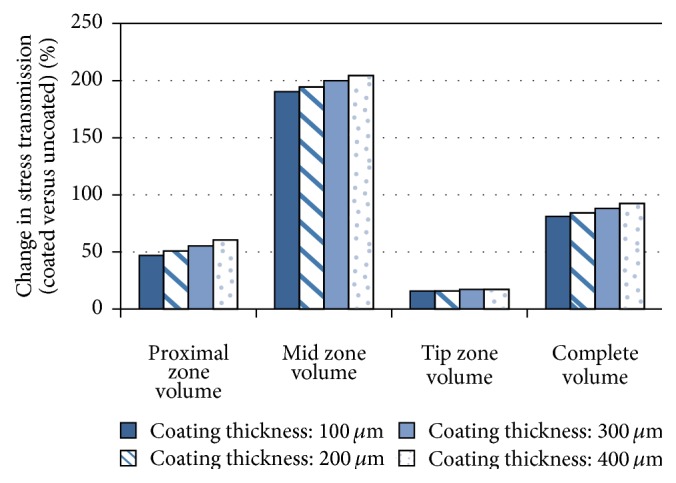
Change in stress transmission to the cancellous bone volume for different thickness of PEEK coatings (see Table 2).

The analysis shown in Figure 7 and Table 2 is based on volumetric average of the effective von Mises stress. It is shown that the gain in load transfer to the bone, for the complete volume of cancellous bone, is 81% for a coating of 100 *μ*m and 92% for a coating of 400 *μ*m. When separating the three zones, the main benefit appears in the proximal and mid zones.

### 3.3. Volumetric Analysis of Femur

**Figure 8:**
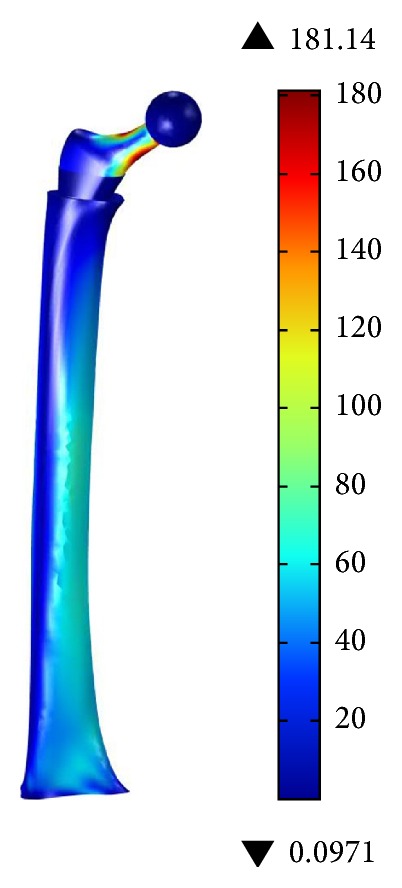
Effective von Mises stress [MPa] for the complete model, using a 400 *μ*m PEEK coating.

Figure 8 shows the results of the Finite Element analysis for the complete model, including the hip implant and the whole femur during a load application of 3,000 N. As expected, the highest stress concentration is on the neck of the hip implant, reaching 180 MPa. It is important to note that the hip implant is hypothetically made of a medical grade titanium alloy that allows these levels of stress. The stress level for the femur is within acceptable levels.

## 4. Discussion

### 4.1. Model Limitations

Some considerations have been taken into account in the model in order to optimize computational resources without affecting analysis fundamentals. The model geometry, specifically on the proximal part of the femur, has been simplified and does not exactly correspond to a femur after THA. Given that the proposed design concept is based on press-fit type implants, a porous surface should be considered. Again, in order to reduce computational load, all analysis were conducted assuming smooth surfaces.

The present study is limited by the use of isotropic mechanical properties for bone, in order to assess the viability of the proposed design concept for the implant. A future more detailed study should consider the actual anisotropic mechanical properties of bone, as well as the variations in bone density due to the remodeling process.

Factors such as the muscle surrounding the femur and changes in force angle while walking are not taken into account in this study. For the purpose of knowing the effect of a coating in the load transmission to the bone, these factors are not essential since human femur while walking is mainly subjected to compression from axial loading. Previous studies have also simplified their models in such a fashion [21–23].

### 4.2. Influence of PEEK Coating and Implant Design on Stress Shielding

Proximal zone presents the higher stress shielding values. During the THA procedure, in order to insert the implant into the femur, cancellous and the intramedullary canal are removed. Then anchoring cement is used to attach the implant to the femur. Since the femur is not a uniform and symmetric geometry, the THA procedure can leave zones with thinner cancellous bone, causing stress peaks as the one seen in the proximal zone in this analysis.

The proposed approach shows an increase of 47% in load transfer in the proximal zone with a PEEK coating with 100 *μ*m thickness, while 400 *μ*m thickness improves by 60% the same indicator. This results compare favorably with the work of Gross and Abel [6], where the increment of von Misses stress in the proximal zone increases 32% with the use of hollow stemmed hip implant.

In a comprehensive review of bone tissue fracture, Doblaré et al. [20] suggest that using the effective von Mises stress is appropriate when considering isotropic bone properties. However, in some studies of this nature, brittle material failure criteria are used: maximum principal stress. In terms of principal maximum stress (compressive stress), the current study was compared to the work of Oshkour et al. [3], where axial load is also taken at 3,000 N. The range of maximum principal stress (compressive stress) found is approximately between 0.3 and 5 MPa (see Figure 5), while Oshkour et al. report a similar range between 0.2 and 4 MPa.

The scope of the present study is to validate the design concept of using a PEEK coating to increases load transmission to the bone. Therefore, most of the analysis conducted here was based on effective von Misses stress as first approach.

In order to have a more robust perspective, further studies should be carried out in order to include muscles such as iliotibial tract. In addition, variation of the angle of the femur while walking should be considered in a more in-depth numerical analysis.

The increased stress at the tip of the implant with PEEK coating, compared to uncoated, might be a concern for periprosthetic fracture. Therefore, a more detailed analysis is required in order to consider an implant redesign of the implant tip or elimination of the PEEK coating in that zone.

The proposed approach opens the possibility to improve mechanical behavior without the need to develop a new implant geometry. With an appropriate coating process, any commercially available implant could be treated in order to improve its performance. The present method of coating a hip implant is not limited to a single implant shape. The relative benefit on the proposed approach on stress shielding will certainly depend on the specific implant geometry, given the wide range of commercially available designs.

The study shows that a significant improvement in the implant performance is reached with a modest amount of polymer coating. In this regard, there are several methods available for coating the hip implant with a polymer. The strongest possibility is to use electrophoretic deposition due to scalability and coating consistency. Electrophoretic deposition is proven method for PEEK deposition in steel [24]; thus other metallic substrates could work in a similar way. One area of concern is the adhesion strength of such a coating over a medical grade titanium stem.

### 4.3. Potential Osseointegration of PEEK Coating

Mineral bone growth, and thus fixation of implant to bone, highly depends on surface roughness and porosity. The use of a PEEK coating on the implant shows the potential to reduce stress shielding, but the question of osseointegration arises. Coatings based on porous hydroxyapatite have been used to promote osseointegration. On the other hand, a number of manufacturing processes are available to generate porous coatings that combine polymers and ceramics. Therefore, this particular issue requires further* in vitro* and* in vivo* studies.

## 5. Conclusions and Future Work

The current study shows that a PEEK coating on a hip implant can improve the load transfer to the bone, minimizing the stress shielding effect and thus prolonging the implant lifespan. The models with coated condition show a significant change in cancellous bone stress, with increase between 81 and 92% in the volumetric numerical analysis.

Further research is required to refine the proposed design concept and develop an appropriate coating process. Additional numerical analysis is required to consider a wider set of loading conditions, as well as anisotropic bone properties. In terms of manufacturing, different coating processes and PEEK formulations require investigation for the hip implant application.

## Figures and Tables

**Table 1:** Material properties used in the FE models for the implanted femur components.

Material	Elastic modulus, *E*[GPa]	Density, *ρ* [g/cm^3^]	Poisson's ratio, *υ*
Cancellous bone	0.155	0.20	0.30
Cortical bone	16.70	1.64	0.30
PEEK 150 XF	3.70	1.30	0.40
Ti-6Al-4V	110.00	4.43	0.33

**Table 2:** 

Coating thickness [*μ*m]	Proximal zone cancellous bone volume		Mid zone cancellous bone volume		Tip zone cancellous bone volume		Complete cancellous bone volume	
	Average stress [MPa]^*∗∗*^	Change coated versus uncoated [%]	Average stress [MPa]^*∗∗*^	Change coated versus uncoated [%]	Average stress [MPa]^*∗∗*^	Change coated versus uncoated [%]	Average stress [MPa]^*∗∗*^	Change coated versus uncoated [%]
Uncoated	1.83	—	0.71	—	2.32	—	1.32	—
100	2.69	47	2.06	190	2.69	16%	2.39	81%
200	2.76	51	2.09	194	2.69	16%	2.43	84%
300	2.84	55	2.13	200	2.72	17%	2.48	88%
400	2.94	60	2.16	204	2.72	17%	2.54	92%

^*∗∗*^Note: effective von Misses stress.
